# Enhanced tumor inhibiting effect of 131I-BDI-1-based radioimmunotherapy and cytosine deaminase gene therapy modulated by a radio-sensitive promoter in nude mice bearing bladder cancer

**DOI:** 10.1093/jrr/rrac075

**Published:** 2022-11-23

**Authors:** Pan Hao, Chunli Zhang, Huan Ma, Rongfu Wang

**Affiliations:** Department of Nuclear Medicine, LuHe Hospital, Capital Medical University, Beijing 101149, China; Department of Nuclear Medicine, Peking University First Hospital, Beijing 100034, China; Department of Nuclear Medicine, Peking University First Hospital, Beijing 100034, China; Department of Nuclear Medicine, Peking University First Hospital, Beijing 100034, China; Department of Nuclear Medicine, Peking University First Hospital, Beijing 100034, China

**Keywords:** neoplasms, gene therapy (GT), 131I, monoclonal antibody, radioimmunotherapy (RIT)

## Abstract

Radioimmunotherapy (RIT) has great potential in cancer therapy. However, its efficacy in numerous tumors is restricted due to myelotoxicity, thereby limiting the dose of radionuclide. To increase tumor radiosensitivity, we incorporated the recombinant lentivirus into the EJ cells (bladder cancer [BC] cells), and examined the combined anti-tumor effects of RIT with ^131^I-BDI-1(^131^I-monoclonal antibody against human BC-1) and gene therapy (GT). The recombinant lentivirus was constructed and packed. The animal xenograft model was built and when the tumor reached about 0.5 cm in diameter, the mice were randomly separated into four groups: (1) RIT + GT: the xenografts were continuously incorporated with the recombinant lentivirus for two days. And 7.4 MBq ^131^I-BDI-1 was IV-injected, and 10 mg prodrug 5-fluorocytosine (FC) was IV-injected for 7 days, (2) RIT: same dose of ^131^I-BDI-1 as the previous group mice, (3) GT: same as the first group, except no ^131^I-BDI-1, and (4) Untreated. Compute tumor volumes in all groups. After 28 days the mice were euthanized and the tumors were extracted and weighed, and the inhibition rate was computed. The RIT + GT mice, followed by the RIT mice, exhibited markedly slower tumor growth, compared to the control mice. The tumor size was comparable between the GT and control mice. The tumor inhibition rates after 28 days of incubation were 42.85 ± 0.23%, 27.92 ± 0.21% and 0.57 ± 0.11% for the four groups, respectively. In conclusion, RIT, combined with GT, suppressed tumor development more effectively than RIT or GT alone. This data highlights the potent additive effect of radioimmune and gene therapeutic interventions against cancer.

## INTRODUCTION

Radioimmunotherapy (RIT) is a process whereby monoclonal antibody-attached therapeutic radionuclides target tumor-based antigens or the tumor microenvironment. In recent years, RIT garnered much attention in treating local and diffuse tumors. In fact, it successfully treated hematological malignancies. For example, radiolabeled anti-cluster of differentiation (CoD) 20 can successfully treat cytosine deaminase (CD) 20-positive non-Hodgkin’s lymphomas [1–2]. Nevertheless, its potency in treating other tumors (i.e. solid tumors) is somewhat disappointing. The main reason being that the radiosensitivity of most solid tumors is lower than that of hematological malignancies. Moreover, the delivery of an effective radionuclide dosage to the solid tumor is often limited by normal tissue tolerance, particularly due to myelotoxicity. Hence, it is essential to increase tumor radiosensitivity such that even small amounts of radioactivity can produce effective therapeutic action against all tumors [3–4].

Currently, it is possible to tweak the ‘genetic profile’ of a tumor to enhance its radiosensitivity [5]. This is generally done by introducing therapeutic genes, with upstream radiation-sensitive promoters (RSPs), to tumor cells. This enables tumor cells to become responsive to radiation, and this results in successful radiotherapy. The concept of employing radio-sensitive genes to improve efficacy of radiotherapeutic drugs is nothing new. In 1994, Boothman demonstrated the regulated expression of therapeutic genes by inserting ‘promoter’ sequences of radiation-inducible genes [6]. More recently, there is more focus on the co-treatment of radio- and gene therapies to fight cancer. Typically, Cytosine deaminase (CD) [7–8], herpes simplex virus thymidine kinase (HSV-tk) [9] and tumor necrosis factor (TNF)-α [10] genes employed as therapeutic genes, and they are placed downstream of early growth response gene (Egr-1) sequences, which serve as the RSP. Earlier studies reported that tumor cells harboring these genes are remarkably sensitive to radiation, even at lower dosage. This indicates that this novel strategy can enhance tumor cell killing, promote tumor developmental delay and augment control of tumor development without affecting healthy tissue, thus, improving the therapeutic ratio.

The Phase I/II clinical trials employed the Ad.Egr. TNF-α plasmid (also known as the TNF erode), along with radiotherapy [10–11], and they observed marked tumor response, without encountering local or systemic toxicity. Egr-1, a synthetic RSP, consists of continuous CC(A/T)6GG sequence (CArG elements) repeats. It was demonstrated to be more sensitive to radiation, relative to the native Egr-1 enhancer [12]. Radionuclide-mediated radiation can strongly activate Egr-1 [13–14]. Therefore, it is speculated that the RIT efficacy will drastically improve once therapeutic genes, with RSPs, are introduced to tumor cells.

Bladder cancer (BC) is the most prevalent urological malignancy in China. BDI-1 is a monoclonal antibody, developed by our hospital, with relative molecular weight of 1.5 × 10^5^ and it targets the human BC cells BIU-87. Immunological investigations revealed that BDI-1 elicits a strong positive reaction in the human BC cell lines BIU-87 and EJ. In a prior investigation, we employed ^131^I labeled anti BC monoclonal antibody BDI-1 to conduct radio immunoimaging and RIT of BC, with encouraging results [15]. To improve tumor radiosensitivity, we constructed a vector harboring the CD gene attached to a synthetic RSP that contained eight upstream CArG repeating elements and a green fluorescent protein (GFP) coding sequence, which served as the reporter gene for monitoring the therapeutic gene expression. Next, we assessed the killing efficiency of ^125^I radiation on the vector-incorporated EJ cells, in the presence of the prodrug 5-FC. In the CD/5-FC suicide system, CD can convert the antifungal drug 5-FC to the cytotoxic drug 5-FU (Fluorourial, 5-fluorouracil). The metabolite of 5-FU in cells inhibits the synthesis of DNA or proteins by inserting DNA or RNA chains, thus playing a cell-killing role. At the same time, 5-FU is also a radiation sensitizer, and its sensitization mechanism may be to reduce the formation of cell nucleic acid, inhibit cell repair and reduce the radiation resistant cells in S phase. Based on our results, we observed a marked therapeutic response in the aforementioned cells versus controls.

In order to increase the radiosensitivity of tumors, we synthesized E8 promoter containing 8 CC(A/T)6GG sequences and linked it to the upstream of the CD suicide gene. We transfected the aforementioned lentivirus E8-codA-GFP LV [16] into the EJ cells in nude mice, and examined the combined anti-tumor effects of RIT with ^131^I-anti BC monoclonal antibody BDI-1 and CD gene therapy (GT).

## MATERIALS AND METHODS

### Cell culture and animal model

The EJ cells were maintained in improved Roswell Park Memorial Institute-1640 (RPMI-1640) with 10% fetal bovine serum (FBS), in a 5% CO_2_ incubator at 37°C, with 45–65% relative humidity. Prior to experimentation, cells were dissociated with trypsin and re-suspended in sterile saline reserve to determine cell concentration [17]^.^ 47 four-week-old female athymic nu/nu mice (20–22 g) were obtained and housed under specific pathogen free (SPF) conditions at the Department of Laboratory Animal Science of our hospital. They acclimatized for 7 days with tap water and a pellet basal diet before the start of the experiments. The temperature was maintained at 23°C ± 2°C, humidity 50–60%, 12 h light/dark cycles. Throughout the experiments, mice were fed with a standard chow diet and tap water. The animal room was cleaned regularly during the holding period. All procedures are carried out in accordance with the Guidelines for Animal. Among mice that will receive tumor xenografts, 27 were evaluated for biodistribution and 20 were examined for therapeutic effect.

The animal xenograft model was generated by the subcutaneous administration of 1 × 10^6^ EJ cells into the left forearm of nude mice. Once the tumor was 0.5 cm in diameter, the nude mice and tumor were evaluated in subsequent experiments. To block the ^131^I uptake by thyroid, mice were fed with 0.5% sodium iodine solution, instead of drinking water, 3 days before and after the treatment.

### Plasmid construction and packaging of the recombinant lentivirus

The plasmid that was incorporated into the BC was constructed as reported previously [18]. Briefly, the CD cDNA was acquired from the pCD2 template using polymerase chain reaction (PCR). The purified PCR products were then ligated to the pGC-FU lentiviral vector containing the GFP gene (Genechem Company). Following cloning and extraction, the plasmid pGC-FU-codA-GFP was obtained. Next, we constructed the RSP E8, harboring eight repeating CArG elements, and ligated it to the pGC-FU-codA-GFP vector to produce the pGC-FU-E8-codA-GFP vector, which was then transfected into 293 T cells. Lastly, we collected the supernatant of 293 T cells, and extracted the recombinant lentivirus E8-codA-GFP LV.

### Infection of the EJ bladder tumor with the recombinant lentivirus

The recombinant lentivirus was provided by the Shanghai Gene Chem Co., Ltd, and it was stable for at least 6 months at −70°C. We generally avoided repeated freeze/thaw cycles, as this can strongly lower transduction efficiency. We also employed polybrene during the process of lentiviral transduction, and used the enhanced infection solution as a diluent for the lentivirus.

Our previous experiment found [8] upon pE8-CD plasmid transfection into EJ cells, the highest transfection efficiency appeared at 48 hours. Therefore, we chose 48 hours after transfection to begin our experiments.

The lentiviral transfection was done as follows: we removed the recombinant lentivirus from the −70° refrigerator, and placed it on a water bath at 37°C. Next, 5 × 10^6^ TU/ 25 uL lentivirus was administered directly to the tumor of the RIT + GT and GT mice, at four points around the tumor. Subsequently, 25 uL of the enhanced infection solution and 1 uL of polyene was administered via intratumor injection in both groups. The above steps were repeated at the same time for two consecutive days.

### Preparation of the ^131^I labeled BDI-1

The antihuman BC monoclonal antibodies BDI-1 was provided by the Hangzhou Jiu yuan Gene Engineering Co., Ltd. BDI-1 was labeled with ^131^I using the chloramine-T (Ch-T) procedure. 150 ug BDI-1 was dissolved in 0.5 mol/L phosphate buffer (PB) (50 uL, pH 7.4) prior to the addition of the Na^131^I solution (30 uL, 200 MBq). Subsequently, fresh Ch-T (10 uL, 1.5 μg/ uL) was added to the mixture, which was then vibrated for 2.5 min at room temperature, followed by purification by Sephadex G-25. Labeling yield and radiochemical purity were assessed via paper chromatography on Xin Hua paper (Hang Zhou Xin Hua Paper Industry Co., Ltd, China) using n-Butyl alcohol: ethanol: ammonium hydroxide 0.5 mol/l = 5:1:2 (v: v: v) as the developing solvent.

### Biodistribution studies of ^131^I-BDI-1 in mice with BC

Twenty-seven nude mice carrying human BC were randomly separated into nine groups of three, and ^131^I-BDI-1 (222 KBq in 0.1 mL/ mouse) was injected into the tail vein. Animals were sacrificed at 0.25 h, 0.5 h, 1 h, 3 h, 6 h, 18 h, 24 h, 48 h and 72 h after injection. Organs of interest were extracted and weighed, and ^131^I radioactivity was recorded using a γ-counter. In terms of the bladder, we determined the percentage of injected dose in the urine. In terms of all other tissues, the radiotracer uptake was $\mathrm{computed}\ \mathrm{as}:\%\mathrm{ID}/\mathrm{g}=\frac{\mathrm{count}(\mathrm{tissue})-\mathrm{count}(\mathrm{background})}{[\mathrm{count}(\mathrm{standard})-\mathrm{count}(\mathrm{background})]\times\mathrm{tissue}\ \mathrm{weight}(\mathrm{g})}\times100\%.$

### Tumor therapeutic studies

Twenty nude mice with human bladder carcinoma were arbitrarily separated into four groups (*n* = 5), namely, RIT + GT (RIT combined with GT), RIT alone, GT alone and control groups. The xenografts of the RIT + GT and GT mice were infected with the lentivirus vector, as described above (see the ‘infection of the EJ bladder tumor with the recombinant lentivirus’). Meanwhile, the RIT and control mice received direct tumor injection of 51 uL 0.9% normal saline. On the third day after intratumor injection, 7.4 MBq ^131^I-BDI-1 was IV-injected into each RIT + GT and RIT group mice. Next, 10 mg of the prodrug 5-FC was dissolved in 400 uL of 0.9% normal saline, and the solution was injected intraperitoneally into each mouse belonging to the RIT + GT and GT groups, once for 7 consecutive days. Meanwhile, 400 uL of 0.9% normal saline was intraperitoneally injected into the RIT and control mice [19].

The long and short diameters of the tumor were measured every day during the first week of treatment, and every other day after the first week of treatment [23]. The treatment period lasted 28 days. Each value was represented as the mean and SD of five animals. The tumor volume was computed as *V*=*ab*^2^/2, whereby, *V* was the tumor volume; a and b represented the long and short tumor diameters, respectively.

The mice were sacrificed, and the tumors were extracted and weighed 28 days after the treatment. The tumor growth inhibition rate was computed as: (tumor weight of untreated mice – tumor weight other mice)/ tumor weight of untreated mice × 100%. If during treatment, a mouse died, the corresponding body and tumor mass were immediately measured [17].

### Statistical evaluation

The results were analyzed using SPSS-23.0, and are presented as $\overline{X}\pm s.$ Single factor analysis of variance was employed to distinguish differences in the tumor volume, tumor weight, body weight and tumor growth inhibition rate among the different groups. *P* value < 0.05 was the significance threshold.

## RESULTS

### Radiolabeling of ^131^I-BDI-1

The radiolabeling efficiency of ^131^I-BDI-1 was 65.31 ± 2.43%, and the radiochemical purity was > 90% [15].

### Tumor morphology

**Fig. 1:**
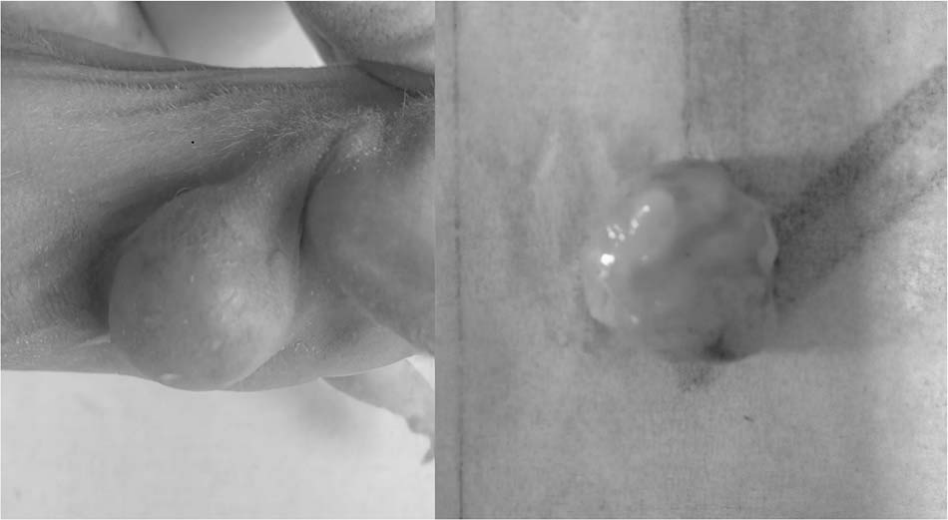
The tumor appearance.

Following 5–7 days of tumor cell incubation in nude mice, we observed subcutaneous nodules, about 1 mm in diameter, at the injection site. The nodules reached about 5 mm at 4 weeks. The tumor surface showed obvious vascular distribution. Following the experiment, upon extraction of the tumor specimen, we observed a complete capsule, with no obvious blood vessels (Fig. 1).

### Radiobiological distribution investigation

The calculated radioactivity uptake rate per gram of tissue (%ID/g) of tumors and major tissues/organs are presented in Table 1. A comparison of the radioactivity of tumor-bearing mice injected with ^131^I-BDI-1(%ID/g, *n* = 3,$\overline{X}\pm s$). ^131^I-BDI-1(^131^I -monoclonal antibody against human BC-1);%ID/g (radioactivity uptake rate per gram of tissue)

**Table 1:** Comparison of biodistribution in tumor-bearing mice injected with ^131^I-BDI-1(%ID/g, *n* = 3,$\overline{X}\pm s$)^131^I-BDI-1(^131^I -monoclonal antibody against human BC-1);%ID/g (radioactivity uptake rate per gram of tissue)

Time (h)	Heart (%ID/g)	Liver (%ID/g)	Spleen (%ID/g)	Lung (%ID/g)	Stomach (%ID/g)	Kidney (%ID/g)	Intestinal (%ID/g)
0.25	4.86 ± 0.12	4.51 ± 0.13	3.24 ± 0.14	5.4 ± 0.02	2.08 ± 0.01	4.6 ± 0.07	2.12 ± 0.01
0.5	5.5 ± 0.04	5.88 ± 0.07	3.5 ± 0.08	5.09 ± 0.01	1.72 ± 0.04	3.96 ± 0.12	1.83 ± 0.04
1	3.75 ± 0.05	4.25 ± 0.01	3.46 ± 0.3	4.69 ± 0.03	1.35 ± 0.01	3.25 ± 0.08	1.65 ± 0.01
3	3.91 ± 0.01	3.86 ± 0.01	2.42 ± 0.01	3.84 ± 0.02	1.01 ± 0.05	2.91 ± 0.1	1.03 ± 0.02
6	3.38 ± 0.13	3 ± 0.02	2.13 ± 0.08	3.54 ± 0.01	0.89 ± 0.01	2.28 ± 0.03	0.91 ± 0.01
18	2.6 ± 0.01	2.14 ± 0.05	1.79 ± 0.01	2.58 ± 0.01	0.70 ± 0.02	1.78 ± 0.04	0.80 ± 0.09
24	1.16 ± 0.06	1.39 ± 0.03	1.2 ± 0.01	2.01 ± 0.04	0.61 ± 0.02	1.03 ± 0.04	0.72 ± 0.02
48	1.05 ± 0.05	0.87 ± 0.01	1.15 ± 0.03	1.67 ± 0.02	0.48 ± 0.02	0.62 ± 0.01	0.21 ± 0.03
72	0.85 ± 0.02	0.66 ± 0.02	0.94 ± 0.01	1.05 ± 0.01	0.74 ± 0.02	0.68 ± 0.01	0.25 ± 0.02
Time (h)	Bladder (%ID/g)	Bone (%ID/g)	Muscle (%ID/g)	Tumor (%ID/g)	Blood (%ID/g)	Brain (%ID/g)	Skin (%ID/g)
0.25 h	6.31 ± 0.12	1.26 ± 0.09	3.08 ± 0.15	1.65 ± 0.14	10.13 ± 0.37	1.06 ± 0.17	2.72 ± 0.33
0.5 h	7.34 ± 0.11	1.92 ± 0.03	2.72 ± 0.04	2.11 ± 0.09	9.72 ± 0.07	0.92 ± 0.38	2.62 ± 0.23
1 h	7.05 ± 0.02	2.79 ± 0.11	1.35 ± 0.01	2.85 ± 0.12	8.91 ± 0.22	0.79 ± 0.06	2.4 ± 0.13
3 h	6.05 ± 0.01	2.55 ± 0.03	1.01 ± 0.02	2.91 ± 0.01	6.83 ± 0.01	0.55 ± 0.03	2.13 ± 2.57
6 h	5.13 ± 0.11	2.54 ± 0.04	0.79 ± 0.11	2.97 ± 0.15	5.46 ± 0.39	0.54 ± 0.03	1.61 ± 0.23
18 h	4.89 ± 0.04	1.85 ± 0.01	0.70 ± 0.09	3.19 ± 0.02	4.53 ± 0.01	0.35 ± 0.07	1.4 ± 0.3
24 h	4.2 ± 0.02	1.01 ± 0.09	0.81 ± 0.01	3.08 ± 0.02	4.41 ± 0.01	0.52 ± 0.01	0.98 ± 0.02
48 h	3.76 ± 0.01	0.76 ± 0.01	0.45 ± 0.02	3.58 ± 0.01	4.03 ± 0.01	0.4 ± 0.02	0.61 ± 0.02
72 h	2.26 ± 0.01	0.28 ± 0.02	0.5 ± 0.02	3.77 ± 0.02	3.81 ± 0.01	0.21 ± 0.02	0.3 ± 0.01

At different time phases following ^131^I-BDI-1 injection, the probe aggregated primarily in the heart, liver, kidneys and bladder and then the tumor. The radioactive uptake was obvious in these major organs and tumor, and as time went on, the radiation count decreased by degrees. The specific ^131^I-BDI-1 uptake in the tumor tissue increased after injection, and remained at a relatively high level for at least 72 h. Its predominant expression in the kidneys indicated the dominant renal-urinary clearance of this imaging agent. Moreover, the blood data revealed that the ^131^I-BDI-1 was characterized by a slow blood clearance, and approximately 3.81% ID/g remained after 72 h.

The calculated % ID/g was high in the spleen, stomach, intestine, but the values decreased over time. Hence, the ratio of T/NT accumulation after ^131^I-BDI administration increased as time elapsed, and reached a maximum at the 72 h time point. Lastly, the ratio of tumor-to-muscle (T/M) and tumor-to-blood (T/B) also peaked at 7.54 and 0.99, respectively, at the 72 h time point.

### The therapeutic efficacy in different groups

**Fig. 2:**
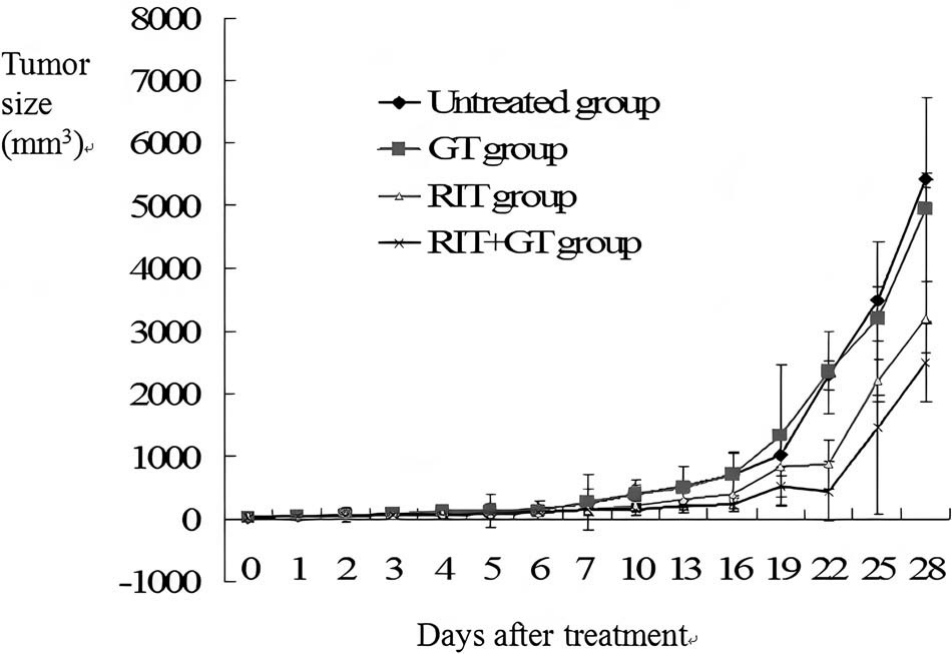
The tumor growth curve in different groups (GT, gene therapy; RIT, radioimmunotherapy).

Figure 2 illustrates the tumor volumes of different groups at different time points after treatment. We next assessed the tumor growth curves of all mice by measuring the tumor volume, as reported earlier. Based on our analysis, the tumor growth rate of the GT and untreated group mice were considerably elevated than the RIT + GT and RIT group mice. Moreover, between the RIT + GT and RIT groups, tumors developed slower in the RIT + GT group, as opposed to the RIT group. So, the tumor growth was stunted in the RIT + GT mice, followed by the RIT mice. There was also rapid tumor growth in the four groups 7 days after, especially the GT and untreated group mice. Tumor growth in the untreated group were not inhibited at all.

No mice died during the course of our 28-day treatment. During the treatment, we observe the health status of the mice, and found that there were no significant differences in the hair color, skin status, activity, reaction sensitivity, diet and water intake between the combined treatment group and the tumor mice in the single treatment group, and there was no abnormal eye, ear, nose and perineal secretions. At the end of treatment, there was no difference in body weight after tumor removal. We did not find more adverse events in the combined treatment group. However, due to the tumor burden, the mice in the untreated group were emaciated, lost weight, had decreased appetite and water intake and moved slowly. Moreover, the body weight of the mice in the untreated group was different from that in the other three groups. Due to the imperfection of the experiment, peripheral blood images (red blood cell [RBC], hemoglobin [HGB], white blood cell [WBC], platelet [PLT]), bone marrow nucleated cell count, heart, liver and kidney functions of mice were not measured. It is expected that the relevant experimental and test data will be improved in the future experimental process. During the fourth week, the surface of the tumor in the RIT + GT and RIT mice appeared necrotic. The tumor volume and weight after 28 days of treatment are listed in Table 2.

**Table 2:** The tumor volume and tumor weight of different groups (RIT, radioimmunotherapy; GT, gene therapy)

**Group**	**Number**	**Tumor volume (mm** ^**3**^**)**	**Tumor weight (mg)**	**Inhibition rate (%)**
RIT + GT	5	2491.10 ± 628.72	998.48 ± 361.87	42.85 ± 0.23
RIT	5	3201.39 ± 569.25	1259.33 ± 329.20	27.92 ± 0.21
GT	5	4952.80 ± 1788.90	1737.17 ± 522.01	0.57 ± 0.11
Untreated	5	5412.86 ± 112.00	1747.13 ± 437.61	—

Based on our observation, the tumor volume and weight of the RIT + GT mice were the smallest, followed by the RIT mice. Moreover, the tumor size was obviously smaller in the RIT + GT and RIT mice, compared to the GT and Untreated group mice following 7 days of treatment (P < 0.05). In addition, there was marked differences in the tumor weights of the GT and untreated mice, as well as the RIT + GT and RIT mice (P < 0.05). The RIT + GT mice also exhibited significantly smaller tumors than the RIT and GT mice (P < 0.05). The tumor inhibition rates after 28 days of treatment were 42.85 ± 0.23%, 27.92 ± 0.2 1% and 0.57 ± 0.11% for the RIT + GT, RIT and GT mice, respectively.

## DISCUSSION

Upon exposure to ionizing radiation, RSPs become active, and promote the expression of downstream genes. Introducing suicide genes, with an upstream RSP, into tumor cells is a promising strategy for anti-tumor GT that combines radiation or radionuclide therapy. CD gene encoding for CD is known to convert nontoxic prodrug 5-FC into cytotoxic 5-FU, which serves an essential role in tumor therapy [20]. Multiple reports suggested that the RSP-based therapeutic gene expression markedly enhances radionuclide therapy [21].

Combining gene and radionuclide therapies has revolutionized the treatment of malignant tumors. The target genes that are generally employed during radiotherapy include p53, Survivin, sKDR, mda2–7, 94/gp96TRAIL, VEGF, CD/TK and so on. Suicide genes possess a powerful bystander effect, in addition to converting nontoxic precursor drugs into cytotoxic drug killer cells [18]. Therefore, they are readily used in treating brain glioma, kidney cancer, lung cancer, cervical cancer and other malignant tumors [20–21].

In order to enhance the tumor therapeutic efficiency of RIT, we attempted to enhance tumor radiosensitivity by infecting tumor cells with the recombinant lentivirus containing a therapeutic gene downstream of an RSP. In our previous study, we constructed a recombinant lentivirus E8-codA-GFP, which included a synthetic RSP E8, with eight CArG elements (CC(A/T)6GG sequence), CD suicide gene and GFP gene reporter. Upon infection of EJ BC cells with the recombinant lentivirus, the cells were irradiated with 37, 74 and 148 kBq of Na^125^I aq., in presence of 5-FC [8]. Using the 3-(4,5-dimethylthiazol-2-yl)-2,5- diphenyltetrazolium bromide (MTT) assay, we demonstrated remarkable killing ability of this novel anti-tumor therapy. Here, we assessed the therapeutic outcome of RIT on tumor xenografts, infected with the recombinant lentivirus E8-codA-GFP in nude mice.

To estimate the radioactivity delivered to tumor tissues, we assessed the biodistribution and retention of ^131^I-BDI-1 in nude mice carrying the EJ BC xenografts. Our results revealed that ^131^I-BDI-1 effectively targeted human BC EJ, and remained within the tumor for a long period of time.

Our previous results involving RIT with ^131^I-BDI-1 monoclonal antibody on EJ bladder revealed partial tumor growth inhibition at a radioactivity of 7.4 MBq. So, we selected this radioactivity for subsequent experiments to examine whether the tumor growth inhibition effect can be enhanced. Following incorporation of the CD gene and RSP E8 into tumors, followed by the subsequent ^131^I-BDI-1 monoclonal antibody treatment, the tumor developments were significantly inhibited. The tumor inhibition rate at 28 days post therapy was 42.85 ± 0.23%, which was significantly higher than the RIT with ^131^I-BDI-1 alone, with a tumor inhibition rate of 27.92 ± 0.21%, and the transfection of the therapeutic gene only into tumor cells, without radiation with a tumor inhibition rate of 0.57 ± 0.11%. Our results indicated that the RIT efficiency can be markedly enhanced by the introduction of a therapeutic gene via an RSP. The tumor inhibition rate of the therapeutic gene incorporation within the tumor, without radiation, was only 0.57 ± 0.11%, which indicated that the basal gene expression was relatively low. These results suggested that the therapeutic gene-based damage to normal tissues may be minimal with this treatment modality.

## CONCLUSION

Our study investigated a novel modality of RIT, combined with GT. Although the tumor inhibition rate was not sufficiently high to achieve an optimal tumor therapeutic level, the results were still encouraging, as it suggested that the potential therapeutic efficacy can be obviously enhanced by elevating the ^131^I-BDI-1 dosage, while optimizing the 5-FC dose. Further research involving this treatment modality is currently underway.

Studies on ionizing radiation combined with radiosensitivity promotors to regulate the therapeutic effects of therapeutic genes on tumors have made progress in many aspects, but most of these studies use external irradiation for induction, and only a few researchers use radionuclide internal irradiation to activate promotors to regulate downstream genes. Hong *et al.* [22] found that 125I-labeled 5-iodine-2 ‘-deoxy riboside (125I-UDR) could induce exogenous EGR-1 gene promoter to activate the expression of β -galactosidase in glioma cells in vivo. 125I-UDR and thymine similar structure, can be incorporated into the synthetic DNA molecules, directly lead to DNA chain breakage, termination of DNA replication, inhibit cell reproduction. In addition, radionuclide internal irradiation therapy combined with GT also includes the accumulation of radionuclides into tumor cells by introducing foreign genes to express proteins with special functions, and then play the targeted killing effect of radionuclides on tumor cells. At this stage, NIS (sodium iodide symporter) gene in normal thyroid tissue is widely used.

Radiosensitivity promoter Egr-1 regulates the ‘CD suicide’ gene in combination with 131I-BDI-1 monoclonal antibody, which plays an additive effect in RIT, and its therapeutic effect is superior to that of monotherapy. These findings reveal that 131I-BDI-1 has great application potential in tumor therapy, laying a foundation for the transformation of 131I-BDI-1 from basic research to clinical application.

Radionuclide GT combined with RIT is one of the main directions of tumor therapy development [22]. Using single anti-tumor specific antigen or the fragment as the carrier of radionuclides, through the antigen antibody specificity combined with carry radioactive nuclide targeting to tumor cells, and induced radiation sensitivity promoter start its downstream suicide gene expression, and then play a radioactive nuclide in radiation therapy and GT for the double role of tumor cells, and the bystander effect of the suicide system. This combination therapy can regulate gene expression and simultaneously kill primary and metastatic tumor cells, which has great potential.

## FUNDING

Department of Nuclear Medicine, LuHe Hospital, Capital Medical University, Beijing, China

## CONFLICT OF INTEREST

We declare that we have no conflict of interest.
